# Dropsy of the Amnion

**Published:** 1875-02

**Authors:** J. C. Bishop

**Affiliations:** Middleport, Ohio


### DROPSY OF THE AMNION.

BY J. C. BISHOP, M.D., OF MIDDLEPORT, OHIO.

*Read before the Meigs County Medical Society, November 23, 1874.*

October 6th, 1873, was called to attend Mrs. S—— in her third confinement. Patient has two healthy, robust children; is large, muscular and well developed; æt. thirty-four. She informed me that her former labors had been easy and natural, and on the present occasion there seemed to be nothing unusual, save the excessive enlargement of the abdomen, which she had associated with the idea of twins, and the peculiar character of the pains, which she declared "did not seem to do any good;" had not felt motion for a week, and for three months had experienced great difficulty in breathing; could not lie down for fear of smothering. She had, during the first few weeks of gestation, an attack of acute articular rheumatism, which yielded readily to the usual alkaline treatment; subsequently, however, there existed for several months an anasarcous condition of the lower extremities. Upon examination, per vaginum, I found the os fully dilated, the amniotic membranes protruding with the fœtal head high up, and reached with difficulty; were unusually firm, and were ruptured at once, the waters coming with a gush, and continuously, until the bed was thoroughly deluged. It continued to flow until passing through the bed to the floor, ran across the room and stood in puddles, and until the head became fairly engaged; even then, by keeping the head pressed against the pubes, with each pain the fluid came away, until I am satisfied, although I had no means of collecting it, that at least two gallons of water passed. The head engaged as soon as the uterus, emptied of this excessive amount of fluid, could come in contact with the fœtus, and labor terminated quickly and naturally, the patient being delivered of a dead male child at full term, decomposition having evidently commenced some days before, as evidenced by the partially denuded and darkened surface. The patient convalesced rapidly, was soon attending to her usual avocations, and, with the exception of a slight facial neuralgia, which yielded readily to quinia and morphia, has since enjoyed excellent health.

This case is introduced as a sort of preface to the subject under consideration, the pathology of which is at present but imperfectly understood, and at the same time opens up an interesting question in uterine pathology.

In the consideration of dropsy of the amnion, it will not be out of place to glance at the amnion in its relations to the uterus, and "en passant" to the foetus; the amount of liquor amnii in health and disease; the conditions producing it in morbid quantity, with diagnosis and treatment. You are acquainted with the physiology of the subject throughout the early development of the fœtus up to the time of the formation of the amniotic cavity, and until the outer lamina of the amniotic fold comes in contact with the vitelline membrane and becoming incorporated with its substance, and constitutes the external investing membrane of the ovary. Coincident with the development of the amniotic membrane is observed the development of the allantois, which, proceeding more rapidly than the former, comes in turn to be the most external, and is brought into relation with the external surrounding media; the subsequent obliteration of the cavity of the allantois, and its conversion into a vascular membrane, which afterwards becomes the chorion; later still, the usurpation of these membranes—three in number—by, *first*, the *amnion*, which is internal and continuous with the fœtal integument; and *second*, the *chorion*, which is *external* and in direct communication with the fœtal abdominal cavity.

Separating these two membranes is a semi-gelatinous fluid, which, according to Dalton, is analogous to the vitreous humor of the eye. In the human subject the formation of the chorion is the same as that of the allantois in the lower animals. The amnion, outgrowing the chorion, encroaches upon the cavity separating them until finally they come in contact, and the cavity is obliterated, while the amniotic membrane comes in contact with the uterine surface, and is reflected over the umbilical cord like the finger of a glove. But few, if any, important changes occur either in the substance or texture of the amnion as the development of the fœtus progresses, save that it gradually acquires the characteristics of a serous membrane, being devoid of either openings or vessels.

Having now traced the amnion to its contact with the internal uterine surface, we proceed to the consideration of the amniotic fluid, which is that contained within the amniotic membrane completely enveloping the fœtus.

In early pregnancy its density is slight; but before term it becomes viscid and unctious. Its color is sometimes clear like serum, and again of a light yellow or greenish; it is sometimes lacterscent, turbid, and interspersed with yellowish-gray, or even black albuminous flakes. In certain cases it is strongly tinged with yellow when the membranes are ruptured from the admixture of mecorium. It exhales a disagreeable odor, analogous to that of the spermatic fluid, and its taste is slightly saline (Cazeaux). According to the analysis of Vanquelin, 100 parts of liquor amnii contain 98.8 parts of water, 1.2 albumen, hydrochlorate of soda, phosphate of lime, and lime. As to the source of the amniotic fluid, authors are at variance; thus, M. Vulpean regards it as the result of transudation or exhalation, just as in other *mucous* membranes; that it requires no particular canals for its accomplishment, being the result of the purely vital phenomena of imbilition. Burdach claims that it is a secretion from the internal surface of the uterus, and reaches the amniotic cavity by traversing its walls. Others regard it being furnished by the fœtus itself.

Dalton (*Physiology*, p. 650), in speaking of the amniotic fluid, says: "At the same time that an albuminous fluid, the 'amniotic fluid' is exuded into its cavity in constantly-increasing quantity." We are unable to prove from whence it comes, but believe it to be the result of a vital process, and that process is one of secretion, similar in all respects to what takes place in the pleura, for instance, for purposes peculiar to itself, save that in the amnion the process is carried a step farther, for purposes hereafter to be considered.

It would be very difficult to say just how far the accumulation of this fluid might proceed even in health, as few of you have observed the same quantity in any two of your cases of parturition; and to draw a dividing line, and say that *to here* is health, and *to here* is disease, would be at least presumption. However, Cazeaux has placed the limit of a healthy accumulation at three or four pounds, from the fact that somewhere within this amount is usually observed. Following him, then, we shall consider that when the liquor amnii exists in a quantity in excess of four pounds, it may be considered pathological, and is a dropsy of the amnion. Why so much variation should exist in the normal amount of this liquor, we opine, will not be satisfactorily explained without the subject shall be more fully considered than heretofore. We infer that the process of furnishing the liquor amnii is subject to various modifications, by conditions of health hygiene, and mental, moral and, may be, hereditary influences, though we are not prepared to say just how much influence is exerted by each of these causes respectively, nor why we sometimes witness in the same subject almost the two extremes as to quantity of this fluid; yet it is true. Granting that when the liquor amnii exists in excess of four pounds it is pathological, it must not be presumed that the morbid conditions producing it can always be recognized, or that they invariably require treatment; while on the contrary these morbid conditions are seldom recognized, and numerous pregnancies, with this complication, terminate favorably to both mother and child (while the rule seems to be unfavorable to the latter). This condition often exists and produces absolutely no inconvenience to the mother, and is only discovered at the moment the membranes are ruptured. How far the secretion of this liquor amnii may go, is impossible to determine. Baudelocque reports two cases in which there escaped respectively 13 and 32 pints. Cazeaux declares that certain authors have known 40 or 50 pints to exist in the amniotic cavity. M. Duclos gives us a case in which "the abdomen was enlarged beyond measure, and having ruptured the membranes collected 14 pounds, without counting what was lost." Dr. O. A. Battson reports a case wherein "at least a gallon of water was allowed to escape" (*Med. and Surg. Jou., Jan.* 21, 1871), and in my own case there was at least two gallons.

The pathological conditions giving rise to a dropsy of the amnion, are in the present state of our knowledge at least "sub judice," and at best only speculative. Dr. Mercier claims it to be the result of inflammation, and has seen the internal surface of the amnion covered several times by false membranes, and the membrane itself highly injected (Cazeaux). It is ascribed to a constitutional syphilis; a morbid condition or ill development of the fœtus; a general infiltration of the maternal system (Ibid). By Dr. Chesney to an anæmia of the mother (*Med. and Surg. Record, Dec.* 3, 1870).

Without stopping to consider each of these theories in detail, your reporter is of the opinion that, since the amnion is a serous membrane (and we believe it is so considered), it must be subject to the general laws governing such membranes in other situations; hence the conditions which induce a dropsical accumulation in one serous membrane, might, "cæteris paribus," tend to its accumulation in another. A true dropsy, wherever found, is essentially a morbid condition affecting the composition of parts, consisting in an abnormal accumulation of liquid exterior to the vessels, derived from the blood, and is in direct relation to the physical properties of the tissues; the result of a morbid attenuation of the blood serum, from undue hydraulic pressure, etc., and not necessarily the result of inflammation, because true dropsies are not dependent upon inflammation, and the causative condition must be sought elsewhere, and remote from the accumulation of fluid; neither is it as generally observed a disease *per se*, but the result of a morbid condition which exists in causative relation to it. Membranes, endowed with the vital property of secretion, are dependent for a normal and legitimate performance of that function upon the healthy or normal condition of the phenomena of absorption, growth and elimination; and any circumstances or conditions which influence unduly the process of nutrition may, and no doubt does, modify this secreting power, as well as the character of the fluid secreted, by rendering it vitiated, causing it to proceed to excess or even arrest it entirely. Thus the fluid secreted by the pleura, peritoneum, pericardium, etc., being dependent upon the proper conduct of the nutritive process in the normal state, the process being a vital one, would undoubtedly, if the nutrition was impaired, either be secreted in excess or arrested, and if the former, a dropsy is the result; so, when this vital process is impaired by either over-exertion, cold, over-stimulation, hereditary disease, etc., the conditions necessary to the proper distribution of a healthy nutrition and functional activity is interferred with, and disease results either directly or remotely; hence dropsy, or an over-accumulation of fluid in these cavities, result. The same causes, then, operating in such a manner to produce an accumulation of fluid in the pleura, would suffice to produce the same or a similar accumulation within the cavity of the amnion, provided the amnion contained the elements of disease which would induce the morbid process, and might be the result of this perverse nutrition, hydraulic or hypostatic pressure, anæmia, lesions of the general circulation, etc. We are of opinion that the morbid accumulation of water in our own case was the result of an improper nutrition, the result of a rheumatic tendency affecting the general circulation, and thus the system at large. Believing the secretion of liquor amnii to be furnished by the mother, we also believe that the conditions producing it in morbid quantity are dependent upon a perverse nutrition. The function of the amniotic fluid has not been satisfactorily demonstrated. That it plays any important *role* in the nutrition of the fœtus, seems to us to need verification, since the fœtal attachments through the placental vessels and cord would readily and rationally account for its nutrition, while the pressure in the fluid of excrementitious material would preclude the idea of its dependence upon the amniotic fluid altogether; if it is a source of nourishment to the fœtus, then those cases in which there is an entire absence of this secretion would insure either a malformation or diseased condition of the fœtus, which we are well aware is not the case. It maintains the isolation of the external fœtal parts, before the skin becomes covered with the sebaceous coat, which at a variable period makes its appearance; allows freedom of motion of the fœtus; protects it from undue uterine pressure; prevents the shock of blows from without; enables it at term to assume a position consonant with the laws of gravity; maintains the integrity of the fœtal circulation by preventing pressure on the cord; renders the uniform dilation of the uterine cervix easy; favors, by lubricating the parts, the passage of the child, and renders the necessary application of instruments comparatively easy; is the receptacle for certain of the excretitions during intra-uterine existence. You will very rarely be called upon to diagnose a dropsy of the amnion, and yet it is not always without some difficulty. A differential diagnosis is only required between this affection and ascites during pregnancy—which may be made by attention to the fact, that in the latter affection the fundus uteri is situated deeply within the abdominal cavity, and its borders can be felt with difficulty; in the former, the uterine tumor is extremely well defined, and is in shape nearly spherodical. In ascites during pregnancy, fluctuation is present and easily detected; in dropsy of the amnion, fluctuation is very limited and not readily detected. In ascites during pregnancy, the thirst is great and constant, and the lower extremities and genitalia are generally infiltrated, the urine scanty, turbid and whitish; in dropsy of the amnion, the thirst is slight, lower extremities not infiltrated, urine natural. In ascites during pregnancy, the tumor is more or less transparent, while in dropsy of the amnion, there is no transparency. The extremely rapid development of the abdomen in dropsy of the amnion is, to a great degree, pathognomonic; for we know of no other condition which would cause so rapid an enlargement in so short a time. Thus, at five or six months, the enlargement is fully equal to an ordinary uterus at full term; while this sudden enlargement operates as a sort of shock to the system, the pressure upwards upon the diaphragm occasions dyspnœa and a sense of choking, which, in uncomplicated pregnancy, is hardly noticed, as the development takes place gradually. The effect of amniotic dropsy upon the mother, so far as has been ascertained, other than inconvenience, is almost *nil;* while the life of the fœtus is generally compromised, is prematurely born or malformed. As to treatment, generally little or none is required; yet, should the enlargement become extreme about the seventh or eighth month, or after the fœtus has developed to such a degree as to render it possible to sustain an existence without the uterus, and the mother become threatened seriously with asphyxia, the membranes should be punctured through the os, high up along the side of the uterus, and a portion of the fluid allowed to flow away, and if possible the full time to pass; but if this is impossible, we should feel justified in inducing premature labor, as a matter of safety to the child.

